# Dual-functional transdermal drug delivery system with controllable drug loading based on thermosensitive poloxamer hydrogel for atopic dermatitis treatment

**DOI:** 10.1038/srep24112

**Published:** 2016-04-19

**Authors:** Wenyi Wang, Elaine Wat, Patrick C. L. Hui, Ben Chan, Frency S. F. Ng, Chi-Wai Kan, Xiaowen Wang, Huawen Hu, Eric C. W. Wong, Clara B. S. Lau, Ping-Chung Leung

**Affiliations:** 1 Institute of Textiles and Clothing, The Hong Kong Polytechnic University, Hung Hom, Kowloon, Hong Kong, China; 2 Institute of Chinese Medicine, The Chinese University of Hong Kong, Shatin, New Territories, Hong Kong, China; 3 State Key Laboratory of Phytochemistry and Plant Resources in West China, The Chinese University of Hong Kong, Shatin, New Territories, Hong Kong, China

## Abstract

The treatment of atopic dermatitis (AD) has long been viewed as a problematic issue by the medical profession. Although a wide variety of complementary therapies have been introduced, they fail to combine the skin moisturizing and drug supply for AD patients. This study reports the development of a thermo-sensitive Poloxamer 407/Carboxymethyl cellulose sodium (P407/CMCs) composite hydrogel formulation with twin functions of moisture and drug supply for AD treatment. It was found that the presence of CMCs can appreciably improve the physical properties of P407 hydrogel, which makes it more suitable for tailored drug loading. The fabricated P407/CMCs composite hydrogel was also characterized in terms of surface morphology by field emission scanning electron microscopy (FE-SEM), rheological properties by a rheometer, release profile *in vitro* by dialysis method and cytotoxicity test. More importantly, the findings from transdermal drug delivery behavior revealed that P407/CMCs showed desirable percutaneous performance. Additionally, analysis of cytotoxicity test suggested that P407/CMCs composite hydrogel is a high-security therapy for clinical trials and thus exhibits a promising way to treat AD with skin moisturizing and medication.

The treatment of atopic dermatitis (AD) has been perplexing clinicians and practitioners for decades^1^,^2^,^3^,^4^,^5^. Conventional mainstream medication always involves the use of topical or systematic corticosteroids, which may cause serious adverse effects such as striae, hypertension or even atrophy; the use of corticosteroids is highly not recommended, especially for young children with AD^6^,^7^,^8^. A wide variety of complementary or alternative therapies, e.g., acupuncture, homeopathy, probiotics, autologous blood injection, massage therapy and herbal preparations, etc., have therefore received considerable attention^9^,^10^,^11^, among which textiles functionalized with physicochemical means or nanotechnology is the most typical adjuvant therapy^12^,^13^,^14^. The development of conventional functionalized textiles is proposed on the basis of reduction or elimination of *S. aureus* colonization associated with AD^15^. Silver nanoparticles have thus been intensively investigated for the development of functionalized textiles due to its excellent antibacterial property^16^. It has been reported that such textiles significantly inhibit *S. aureus* growth and are viewed as a promising candidate for AD treatment^17^,^18^. Additionally, zinc oxide nanoparticles and some naturally occurring organic compounds, such as citric acid and borage oil, have also been reported in the functionalization of textiles^19^,^20^,^21^. However, an inevitable problem for these functional textiles is that they cannot rehydrate the skin and may exacerbate the xerodermia of AD patients, even though they showed therapeutic effects to some extent^22^. For this purpose, wet-wrap dressing is thus considered a satisfactory alternative to functionalized textiles for moisturizing the skin and controlling the transepidermal moisture loss^23^,^24^. Unfortunately, wet-wrap dressing mainly serves as a moisture reservoir and its prolonged use normally leads to an uncomfortable feeling^25^. Consequently, the therapeutic effect of wet-wrap dressing is apparently limited and can be negligible unless it is used in combination with some medications^26^,^27^. Inspired by this, we are prompted to develop a transdermal drug delivery system with dual-functions, i.e., moisture and drug supply (Fig. 1), which may be used for development of functionalized textiles which can release the drug in a controlled manner.

Hydrogel offers an ideal solution for developing this preconceived transdermal drug delivery system with dual-functions. Hydrogels are hydrophilic three-dimensional polymeric networks capable of absorbing a large amount of water or biological fluids^28^. The high moisture content renders hydrogels compatible with most living tissues and thus facilitates widespread application in biomedical and pharmaceutical areas^29^,^30^. Thermo-sensitive hydrogel exhibits a free-flowing sol at low temperatures but becomes a gel at body temperature, which facilitates administration and accessibility when applied in drug delivery systems^31^,^32^. Poloxamer 407 (Pluronic F127) is one of the most typical thermosensitive polymers and has been approved by the FDA. Poloxamer 407 can self-assemble into micellar structures and form hydrogels under certain conditions (Fig. 2e). The micellization results from the dehydration of hydrophobic PO blocks and the resultant micelles are spherical with a dehydrated polyPO core and an outer shell of hydrated swollen polyEO chains^33^,^34^. A high drug loading can therefore be achieved simply by incorporating hydrophilic drugs into the micellar structures. Also, bioadhesive polymers such as cellulose derivatives are normally added to enhance the poor bioadhesive property of poloxamer-based hydrogels^35^. This, typically, not only contributes to increase of bioadhesive property of the system, but also improves the surface morphology structure, i.e., porosity, which is demonstrated visually in the present study.

Therefore, we hypothesize that the proposed dual-functions transdermal drug delivery system can be achieved via poloxamer-based hydrogel. For providing workability of the concept, Poloxamer 407/Carboxymethylcellulose sodium (P407/CMCs) composite hydrogel containing a model traditional Chinese medicine, namely, Cortex Moutan (CM) extract was prepared. The drug-loading hydrogel was sufficiently investigated in terms of gelation transition behavior, rheological property, *in vitro* drug release, percutaneous delivery behavior and cytotoxicity test, to provide the groundwork for clinical trials in AD treatment.

## Results

### Phase transition analysis

**Figure 2:**
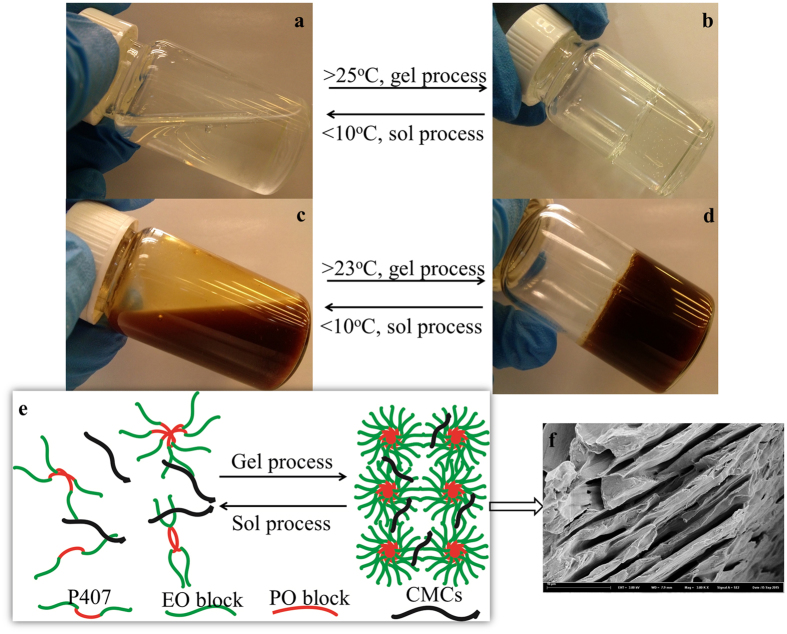
Micrographs to show sol-gel transition of P407/CMCs composite hydrogel (**(a,b)** representing blank hydrogel P200; **(c,d)** representing drug loaded hydrogel PC204); the sol-gel transition is elucidated by schemati cdiagram **(e)**; and the FE-SEM cross-section image **(f)** (3000x) to illustrate the porous structure caused by ordered spherical micelles.

**Figure 3:**
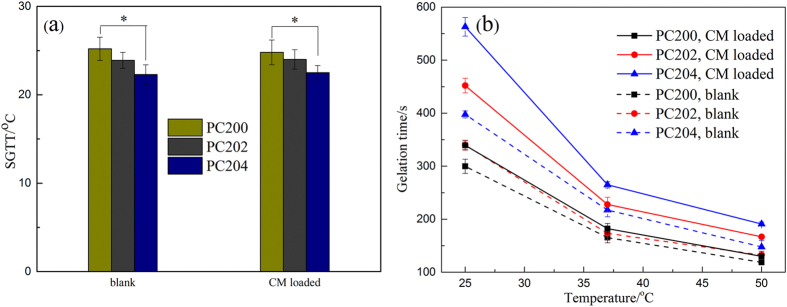
SGTT **(a)** and gelation time **(b)** of P407/CMCs hydrogels as a function of the temperature measured by “tube inversion method” (n = 5, *p < 0.05).

Phase transition properties of composite hydrogel formulations were measured using tube inversion by observing the flowability of P407/CMCs solution. As shown in Fig. 2, the composite hydrogel of P407/CMCs exhibited remarkable reversible sol-gel transition properties. The gelation mechanism can be interpreted by dehydration of PO block and intermicellar interaction (Fig. 2e)^36^. The presence of hydrophilic CMCs can strengthen the hydrophilic chain of the system, thereby reducing the SGTT (Fig. 3) and enhancing the storage modulus of P407/CMCs composite hydrogel. Figure 3 clearly shows that the SGTT slightly decreased on addition of CMCs and accordingly, the gelation time saw a noticeable increase. As expected, there was a marked decline in the gelation time as the temperature increased. Likewise, compared to blank hydrogel, the gelation time of CM loaded hydrogel also dropped appreciably. However, CM was not found to significantly alter the SGTT (Fig. 3a).

### Surface morphology analysis

**Figure 4:**
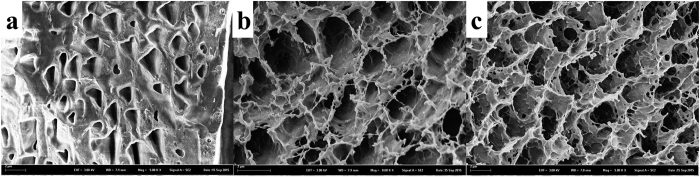
SEM images of PC200 **(a)** (3000x), PC202 **(b)** (3000x) and PC204 **(c)** (3000x) hydrogel.

FE-SEM morphology of varying formulations of lyophilized P407/CMCs composite hydrogel is depicted in Fig. 4. As expected, surface morphology of P407 was altered with the addition of bioadhesive polymer (CMCs), i.e., an apparent increase in the porous structure (Fig. 4b,c). It is evident that the porosity is positively correlated with concentration of CMCs, i.e., high concentration results in high porosity. This porous structure is expected to afford excellent drug-loading capacity and controlled release property to the composite hydrogel.

### Rheological behavior

**Figure 5:**
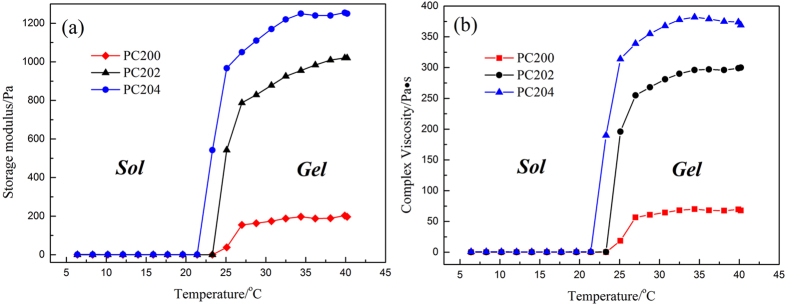
Storage modulus and complex viscosity versus temperature curves of various formulations of P407/CMCs hydrogel.

As seen in Fig. 5, the rheological characteristics of P407/CMCs binary composite hydrogel are temperature-dependent. With increasing temperature, various hydrogel formulations exhibit a consistent alteration in the complex viscosity and storage modulus, both of which increase sharply at the gelation temperature. The highest storage modulus and complex viscosity were seen in PC204. Analogous to phase transition analysis, rheological analysis also shows that the presence of CMCs slightly lowers the SGTT but enhances the storage modulus and complex viscosity due to intermicellar interactions and an increase in the effective length of the hydrophilic chain. Considering the abundant porous structure of composite hydrogel formulations with the addition of CMCs, PC204 was the optimal specimen for further experiments.

### *In vitro* cytotoxicity

**Figure 6:**
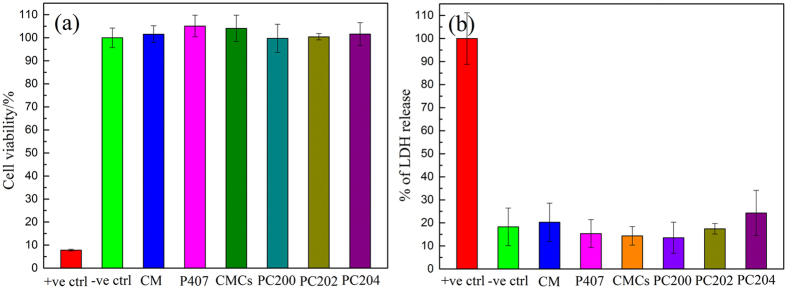
*In vitro* cytotoxicity of different formulations of P407/CMCs composite hydrogel against EpiDerm, (**a**) MTT assay and (**b**) LDH assay.

Figure 6 presents the cytotoxicity analysis of various formulations of P407/CMCs composite hydrogel. Clearly, all formulations are shown to be nontoxic against human epidermal equivalent (EpiDerm). It is shown that P407/CMCs binary composite hydrogel would be safe for clinical practice.

### *In vitro* drug release profile

**Figure 7:**
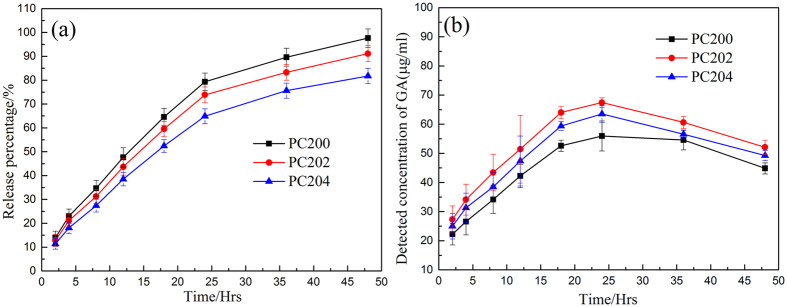
Cumulative release of CM from various formulations of P407/CMCs composite hydrogel ((**a**) release percentage; (**b**) detected concentration of GA). The experiments were performed in triplicate by the dialysis method.

Figure 7 illustrates the profile of *in vitro* release of CM from various formulations of P407/CMCs composite hydrogel. It is clearly observed that over 95% of CM was released from PC200 within 48 h, whereas PC202 and PC204 released less than 90% of CM during the period. The three formulations had a steady release rate over 24 h, after which the release rate gradually decreased. Moreover, PC200 had the highest release rate, while PC204 exhibited the lowest rate, indicating that the addition of CMCs reduced the rate of release of the drug. This was primarily because the presence of CMCs raised the viscosity in the system and the high viscosity served to retard the diffusion of the drug. Additionally, the dialysis method we adopted in the present study allowed permeation of water molecules across the dialysis membrane. Considerable structural changes thus occurred, including alteration of shape and size distribution of the pores, due to progressive swelling of hydrophilic composite hydrogels, resulting in an increase in the tortuosity during the diffusional release of the drug^37^.

### Transdermal drug behavior

**Figure 8:**
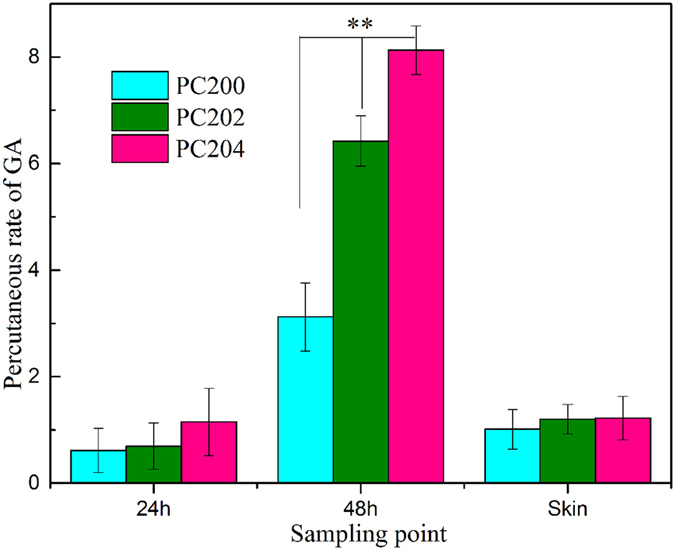
Cumulative release of CM permeated through porcine ear skin following topical application (n = 3, mean values ± SD, **p < 0.01), drug loading of the three formulations was 50%.

The transdermal behaviors of CM extract permeated through porcine ear skin from three formulations of P407 hydrogel were shown in Fig. 8. As shown in Fig. 8, the highest level of drug percutaneous permeation was seen in PC204, both within 24 h and 48 h, whereas the smallest quantity of drug penetrated from PC200 during the same period. This shows that the percutaneous behavior of CM across the skin was enhanced in the presence of CMCs. In the meantime, there were no statistically significant differences (*p* > 0.05) among the three formulations in terms of drug retained in the porcine skin, all of which withheld approximately 1% of GA.

## Discussion

Percutaneous administration is an attractive route for local treatment since several advantages—it does not cause pain, avoids hepatic first-pass metabolism and enhances patient compliance in long term therapy—compared to other forms of drug delivery^38^,^39^. The application of thermo-sensitive hydrogels has gained increasing attention in the development of transdermal drug delivery systems for ages^40^,^41^. Currently, several transdermal delivery products are available in the market^42^. For the treatment of AD, however, to the best of our knowledge, the related literature is scarce, especially in terms of use of hydrogel.

Here, we have developed a thermo-sensitive P407/CMCs composite hydrogel with dual functions, i.e., moisture and drug supply. As shown in Fig. 2, P407/CMCs composite hydrogel is a transparent liquid at low temperature (below 10 °C) and is transformed into a gel after around 3 minutes when the temperature rises to 37 °C (Fig. 2). The gelation time decreases significantly as the temperature increases (Fig. 3). The SGTT for PC200, PC202 and PC204 was less than 25 °C, but PC200 showed the highest SGTT, indicating that the presence of CMCs can slightly reduce the temperature of sol-gel transition. This could be well interpreted by the intermicellar interactions, which become more prominent at higher CMCs concentration^36^. The rheological analysis further confirms the slight diminution in the temperature of gelation transition after adding CMCs (Fig. 5). However, it is worth noting that the viscosity was altered appreciably in the presence of CMCs, and it was found positively related to concentration of CMCs. The reason for this is also intermicellar interactions^36^. Accordingly, the storage modulus also showed a marked increase when CMCs was added. It was shown that CMCs can remarkably change the physical structure of P407 gel, which was further confirmed visibly by the FE-SEM images (Fig. 4). FE-SEM images (Fig. 4) demonstrate that composite hydrogels (PC202 and PC204) exhibit excellent porous structures and increasing the proportion of CMCs leads to high porosity. This is desirable for high drug loading and controlled release. In order to evaluate the release profile and percutaneous behavior, the drug loading in this study was set as 50% (w/w) for all hydrogel formulations. This was far higher than microcapsules reported in our previous study^43^,^44^, also without any loss of active ingredients of herbal medicine (CM) compared with microencapsulation approach. Actually, the excellent physicochemical property, i.e., hydrophilicity, of the composite hydrogel adopted in the present study makes the drug loading highly controllable for the hydrophilic CM extract. This would be very conducive to tailor the loading of CM in the composite hydrogel without considering excessive drug loading and thus causing toxicity.

Furthermore, from the rheological analysis (Fig. 5), the highest viscosity above the critical gel temperature was seen in PC204, which makes it more suitable for application in transdermal drug delivery. High viscosity helps maintain integrity of the hydrogel formulation when applied in transdermal delivery and avoids the flowability. Meanwhile, the property of sol-gel transition below the critical gelation temperature makes it more accessible to prepare hydrogel formulations and hydrogel-coated fabrics.

*In vitro* cytotoxicity was assayed using MTT and LDH method before clinical practice to guarantee safety (Fig. 6). MTT is a yellow tetrazolium salt and can be reduced to a blue colored formazan only in living, metabolically active cell mitochondria^45^,^46^. Therefore, MTT assay is a measure of cell viability. LDH assay, which measures the leakage of LDH (cytoplasmic enzyme) into the extracellular fluid, is a measure of cell membrane integrity^46^,^47^. As seen from Fig. 6(a), the cell viability of all the specimens were nearly 100% and there was no statistically significant difference (*p* > 0.05) between specimens and negative control. A similar phenomenon is also seen in Fig. 6(b). These results showed the non-cytotoxicity of P407/CMCs composite hydrogel formulations. This means that the fabricated P407/CMCs composite hydrogel formulation may be safe for clinical use. It should be noted that, however, the Epiderm we adopted for the present study is a commercially available human epidermal equivalent, which effectively provides a non-animal means to assess dermal toxicology and skin research issues^48^. A growing body of data indicates that Epiderm can thus be used as full replacement of the *in vivo* rabbit skin test, which has been recognized by the ECVAM Scientific Advisory Committee (ESAC)^48^,^49^. Accordingly, it can be concluded that P407/CMCs composite hydrogel is a high-security therapy for clinical use in AD treatment.

**Figure 9:**
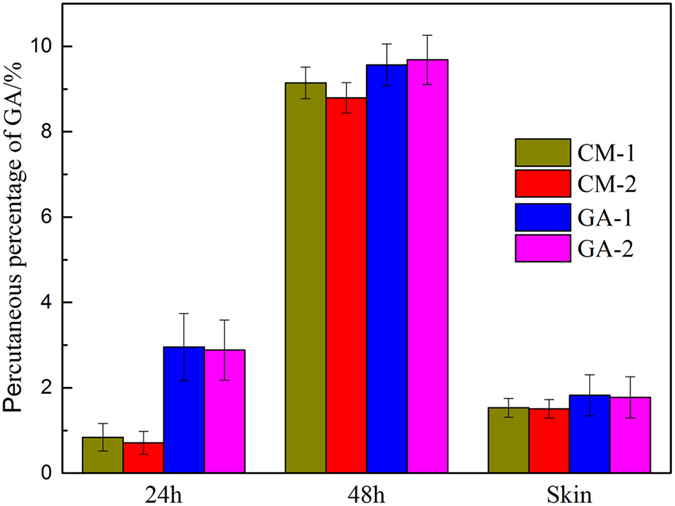
Cumulative release of GA permeated through porcine ear skin (n = 3, mean values ± SD). CM-1 representing CM solution, CM-2 representing CM solution with 4% (w/w) CMCS, GA-1 representing GA solution and GA-2 representing GA solution with 4% (w/w) CMCS, concentrations of CM and GA were identical to PC200, PC202 and PC204.

Next, the profiles of *in vitro* release of CM from three composite hydrogel formulations were investigated by the dialysis method (Fig. 7). Although the addition of CMCs lowered the release rate of CM due to an increase of viscosity of the system, the accumulative release percentage still reached close to 90% (89% for PC202 and 81% for PC204) after 48 h. On the contrary, however, from the transdermal analysis results (Fig. 8), it can be clearly observed that the percutaneous behavior of CM across the porcine skin was significantly enhanced in the presence of CMCs, especially after 48 h. This was further validated by the statistical analysis (*p* < 0.01). The reason, we speculate, may be ascribed to either the penetration enhancing effect of CMCs on GA^39^ or the increasing porosity of the composite hydrogel as CMCs was added^28^,^50^. In transdermal drug delivery, penetration enhancers are commonly used to promote the diffusion of drug through the skin by overcoming the barrier function of stratum corneum^39^. To verify our speculation, an experiment on the ability of CMCs to enhance penetration of GA through the skin was conducted (Fig. 9). In the experiment, the penetration behavior of CM in the presence of CMCs was first investigated. As a control, GA was also tested in terms of percutaneous behavior in the presence of CMCs. The percutaneous analysis showed that the permeation of GA across the skin was not significantly enhanced (*p* > 0.05) with the aid of CMCs, both for CM and GA specimens, indicating that CMCs has no permeation enhancing ability. Hence, an increase in the porous structures of composite hydrogels with the addition of CMCs was the paramount parameter which accounted for enhancement of the penetration behavior of CM.

Taken together, the presence of CMCs noticeably altered the physical properties of P407/CMCs composite hydrogel. The abundant porous structure makes P407/CMCs composite hydrogel serve as an ideal carrier to load hydrophilic drugs with controllable drug loading, and it was shown that such porous structure also facilitated the diffusional release of drugs, thereby promoting transdermal drug delivery. Meanwhile, with the high moisture content, P407/CMCs composite hydrogel may be employed as a promising therapy with dual-functions to provide both moisture and drug for the skin of AD patients. Considering the excellent anti-bacterial properties and anti-inflammatory activities of the herbal drug—CM extract and its major active component GA^51^,^52^,^53^, It is believed that the proposed herbs-containing hydrogel therapy possess a promising clinical application in AD treatment. The clinical efficacy and effectiveness of this proposed therapy will be validated clinically in our future work.

## Conclusions

This study developed a P407/CMCs composite thermosensitive hydrogel with dual-functions of both moisture and drug supply. A series of characterization was sufficiently conducted to evaluate the physicochemical properties of fabricated drug-loading composite hydrogel formulation, which makes it feasible and possible to be potentially applied for clinical trials in AD treatment. FE-SEM images showed that the presence of CMCs can endow the hydrogel formulation of P407/CMCs with excellent porous structures, which was found to facilitate the diffusional release of CM across the skin. The sol-gel transition temperatures of P407/CMCs composite hydrogel can be tailored simply by controlling the addition of CMCs without causing the alteration of the sol-gel transition property. The rheological property, however, showed significant change, with a remarked increase in the storage modulus and complex viscosity. The findings from transdermal studies indicate that P407/CMCs composite hydrogel have desirable percutaneous behavior. From the analysis of cytotoxicity test, it can be concluded that P407/CMCs composite hydrogel formulation may be safe for clinical trials for AD treatment and thus exhibits a promising dual-functional therapy to treat AD.

## Methods

### Materials

Poloxamer 407 (P407, MW = 11500, PEO_101_-PPO_56_-PEO_101_) was purchased from International Laboratory (USA). Carboxymethyl cellulose sodium (CMCs) was obtained from Wako Chemicals. Acetonitrile (HPLC grade) and Dulbecco’s phosphate-buffered saline (DPBS) were provided by Thermo Fisher Scientific. Cortex Moutan (CM) aqueous extract was supplied by the Institute of Chinese Medicine (ICM), the Chinese University of Hong Kong. All other reagents were of analytical grade and used as needed.

### Preparation of P407/CMCs composite hydrogel

The fabrication strategy of P407/CMCs composite hydrogel was “cold method” as described by Schmolka^54^. Briefly, a predetermined amount of P407 was added into CM solution in an ice bath with the final P407 concentration at 20% (w/w) under constant magnetic stirring. The dispersion was then refrigerated until P407 was completely dissolved and a clear solution was obtained. Afterwards, CMCs was added into P407 solution to form binary composite hydrogel under constant stirring with the final CMCs concentrations at 2% and 4% (w/w), respectively. As control, P407 hydrogel without CMCs was prepared and labelled as PC200 for further discussion. Likewise, P407 hydrogels (20%, w/w) with the final concentrations of CMCs at 2% and 4% (w/w) were labelled as PC202 and PC204, respectively.

### Characterization of P407/CMCs hydrogel

#### Sol-gel transition temperature (SGTT) and gelation time (GT)

The tube inversion method was used to measure the sol-gel transition temperature and gelation time. Briefly, 8 ml of each formulation was tested in glass vials (27.5 × 57 mm, clear glass) and heated slowly from 10 °C to 40 °C at a rate of 1 °C/min in a water bath. At each temperature point, the sample was equilibrated for 10 min, and flowability of the samples was observed by tilting the vials. The temperature at which the liquid was immobile was recorded as the gelation transition temperature. Gelation time was measured in a similar manner at constant temperatures of 25 °C, 37 °C and 50 °C. Time measurements were initiated when samples incubated in the water bath. The flowability of samples was observed every 30s and the time at which the samples became stationary was recorded as the gelation time.

### Surface morphology

The shapes and surface morphology of vacuum lyophilized P407/CMCs hydrogel were examined by field emission scanning electron microscope (FE-SEM). The samples were fixed with conductive tape on a metal stub, sputter coated with gold under vacuum, and observed at a 3 kV accelerating voltage at a working distance of 8.0 mm using a JEOL 6490 (Hitachi, Japan).

### Rheological behavior

The rheological properties of PC200, PC202 and PC204 were measured using a Physica MCR301 rheometer (Anton Paar, Austria), operated with parallel plates (diameter 25 mm). Silicon oil was used to avoid water evaporation on the outer edge of the sample. During the gelation process, temperature was maintained between 5 °C and 40 °C with a constant heating rate of 1 °C/min. The values of storage modulus (*G′* ) and complex viscosity (η*) were recorded as function of temperature at a fixed frequency of 1 Hz. Gelation temperature (*T*_*gel*_) was determined from the inflection points of the recorded graphs.

### *In vitro* cytotoxicity test

#### Epidermal culture

To investigate the safety performance of prepared hydrogels for further use in clinical trials, cytotoxicity evaluation was conducted using Epiderm Assay Kit, i.e., EpiDerm (EPI-200, MatTek Corporation, Ashland, MA, USA), which is commercially available human epidermal equivalent. The EpiDerm culture comprised of human-derived epidermal keratinocytes, cultured on the standing cell culture inserts (Millipore, Billerica, MA, USA), at the air–liquid interface of which a multilayered and differentiated model of the human epidermis was formed. The EpiDerm culture was preconditioned overnight at 37 °C and 5% CO_2_ in a 6-well plate. Samples and positive control (Sodium Dodecyl Sulfonate, SDS, 5%, w/v) were added to the skin inserts the next day and incubated for 1 hr. Afterwards, all the skin inserts were transferred into fresh medium for 24 hrs for cytokines release (if any) to the medium. Medium was recovered and stored at −80 °C for LDH assay and then the skin inserts were transferred to fresh plates for MTT assay. The non-treated skin insert was used as negative control.

#### Lactate dehydrogenase (LDH) assay – cell membrane integrity test

The cell membrane integrity (cell damage) of the EpiDerm culture was measured by a colorimetric LDH assay (LDH Cytotoxicity Detection Kit, Takara Bio Inc., Otsu, Shiga, Japan). This assay measures membrane integrity as function of the amount of cytoplasmic LDH released into the medium. Cell membrane integrity is expressed as the ratio of the amount of LDH released, per treatment, to the maximum amount of LDH released from the positive control.

#### MTT assay – cell viability test

Skin inserts were transferred to fresh plates with pre-filled MTT solution (MTT was dissolved in PBS at 5 mg/ml and filtered to sterilize and remove a small amount of insoluble residue present in some batches of MTT), and incubated for 3 hrs at 37 °C and 5% CO_2_. Upon completion of incubation, all MTT solution was removed. Skin inserts were then transferred to fresh plates and isopropanol was added to each insert for formazan extraction for 2 hr. Then the spectrophotometric analysis of extracted formazan at 550 nm was carried out in a 96-well plate. Cytotoxicity was expressed as the ratio of the cell viability, per treatment, to the maximum cell viability from the negative control (non-treated skin insert).

### *In vitro* drug release profile

The profiles of *in vitro* release of CM from PC200, PC202 and PC204 hydrogel were investigated by a dialysis method. Formulations with drug loadings of 50% were sealed in a pre-swollen cellulose membrane dialysis bag (3500 molecular weight cut-off) and immersed into 10 ml of DPBS at 37 °C in a water bath shaken at 100 rpm. At designated time intervals, 2 ml aliquots of the release medium were withdrawn and replaced with an equal volume of the medium. Gallic acid (GA), one of the principal active ingredients of CM, was analyzed, for the convenience of discussion. The concentration of GA in the release medium was assayed by a high performance liquid chromatograph (HPLC), Hewlett Packard agilent 1000 series. The mobile phase consisted of acetonitrile and double distilled water/phosphoric acid (99.0/1.0, v/v) and the flow rate was 1.0 ml/min. All of the experiments met the sink conditions and were repeated thrice.

### *Ex vivo* transcutaneous studies

*Ex vivo* drug percutaneous behavior was investigated by using a Franz diffusion cell with a 6.5 ml receptor compartment. The fresh porcine ears were purchased from local butcher. The ear skin was freshly excised and isolated from the ear with a scalpel. The excised ear skin was then mounted between the donor and the receptor compartment of the diffusion cell. The drug loaded hydrogel samples were placed over the skin and covered with paraffin film. The receptor compartment of the diffusion cell was filled with DPBS and constantly stirred magnetically at 300rpm; the temperature was maintained at 37 ± 0.5 °C. The samples were withdrawn at different predetermined time intervals and replenished immediately with an equal volume of fresh DPBS to maintain sink condition. The samples were then analyzed for drug content spectrophotometrically.

### Statistical analysis

The experimental data were analyzed using Origin software (version 9.0), for deriving standard deviation, one-way ANOVA test and Bonferroni test. A p-value of 0.05 was taken as the level of significance and the data were labeled with (*) for *p* < 0.05, and (**) for *p* < 0.01, respectively. Each experiment was conducted in triplicate (n = 3).

## Additional Information

**How to cite this article**: Wang, W. *et al*. Dual-functional transdermal drug delivery system with controllable drug loading based on thermosensitive poloxamer hydrogel for atopic dermatitis treatment. *Sci. Rep*. **6**, 24112; doi: 10.1038/srep24112 (2016).

## Figures and Tables

**Figure 1:**
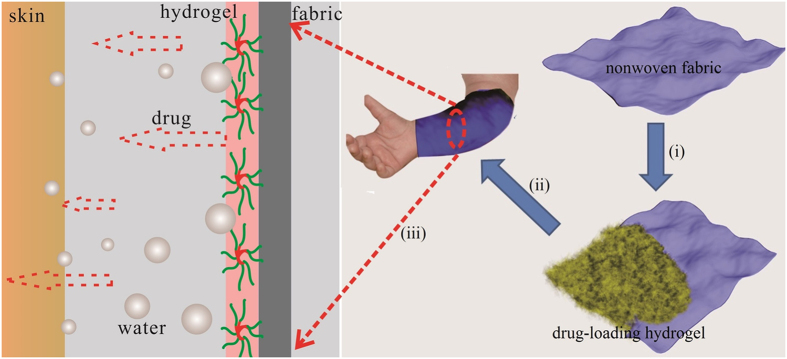
Schematic diagram of hydrogel functionalized fabric with dual-functions; (i) to coat drug-loading hydrogel onto the fabric; (ii) to apply the hydrogel coated fabric to the skin of a patient; and (iii) to show how the coated fabric works with moisture and drug diffusion across the skin.
